# Das biopsychosoziale Verständnis von Gesundheitsstörungen und Beeinträchtigungen bei Kindern mit Fokus auf entwicklungsneurologische Zusammenhänge

**DOI:** 10.1007/s00103-023-03732-1

**Published:** 2023-06-15

**Authors:** Ute Thyen, Juliane Spiegler, Kerstin Konrad

**Affiliations:** 1 Klinik für Kinder- und Jugendmedizin, Universität zu Lübeck, Ratzeburger Allee 160, 23538 Lübeck, Deutschland; 2 Klinik für Kinder- und Jugendmedizin, Universität Würzburg, Würzburg, Deutschland; 3 Sektion Klinische Neuropsychologie des Kindes- und Jugendalters, Klinik für Psychiatrie, Psychosomatik und Psychotherapie des Kindes- und Jugendalters, RWTH Aachen, Aachen, Deutschland; 4 JARA-Brain Institut-II Molekulare Neurowissenschaften und Bildgebung (INM-11), Forschungszentrum Jülich/Aachen, Aachen, Deutschland

**Keywords:** Psychische Gesundheit, Kindergesundheit, Jugendgesundheit, Multiple chronische Erkrankungen, Neurobiologie, Mental health, Child health, Adolescent health, Multiple chronic conditions, Neurobiology

## Abstract

Die Unterscheidung von mentalen (geistigen und psychischen) und körperlichen Gesundheitsstörungen ist aufgrund der Besonderheiten der neurobiologischen Entwicklung bei Kindern und Jugendlichen besonders schwierig. In diesem Übersichtartikel werden zunächst die entwicklungsneurologischen Grundlagen kurz beschrieben. Anhand einiger angeborener oder früh erworbener neurologischer Erkrankungen wird dann aufgezeigt, in welch unterschiedlichem Maß dabei auch mentale Prozesse beeinträchtigt sein können, auch in Wechselwirkungen mit den sozialen Kontextfaktoren. Die Berücksichtigung dieser Aspekte spielt bei der kind- und familienorientierten Beratung und Begleitung eine wichtige Rolle. Das häufige, aber auch interindividuell sehr variable und im Lebenslauf fluktuierende gemeinsame Auftreten von körperlichen, geistigen und psychischen Entwicklungsstörungen verlangt nach einer guten interdisziplinären Kooperation zwischen konservativer und operativer Kinder- und Jugendmedizin und Kinder- und Jugendpsychiatrie.

## Abstract (en)

The distinction between mental (spiritual and psychological) and physical health disorders is particularly difficult due to the special features of neurobiological development in children and adolescents. In this review article, the basics of developmental neurology are briefly described. On the basis of some congenital or early acquired neurological diseases, it is then shown to what extent mental processes can be impaired in interactions with the social context. Taking these aspects into account plays an important role in child and family-oriented counseling and support. The common occurrence of physical, mental, and psychological development disorders, which is also very variable between individuals and fluctuates over the course of a person’s life, requires good interdisciplinary cooperation between conservative and surgical child and adolescent medicine and child and adolescent psychiatry.

## Hintergrund

Die Beziehungen von Geist und Körper, Seele und Leib oder Psyche und Physis zueinander beschäftigen die Heilkunde seit Jahrtausenden. Dabei wird die Frage nach dem Bewusstsein und der Identität des Menschen berührt. Im 19. Jahrhundert erfolgte aufgrund der sehr raschen Entwicklung von neuen Technologien und einer Spezialisierung in der Medizin eine verstärkte Reduktion körperlicher auf zelluläre und physiologische Prozesse. Als Gegenposition zu dieser „Medizin der seelenlosen Körper“ (von Uexküll) entwickelt die Psychosomatik eine Integration von körperlichen und seelischen Zuständen und ein biopsychosoziales Modell von Krankheit und Gesundheit [1]. Wenngleich heute ein weit verbreiteter Konsens in der modernen Medizin besteht, dass jeder Patient ein psychosomatischer Patient sei, spiegelt sich dies wenig in der Gestaltung der Ausbildung, in der ärztlichen fachlichen Spezialisierung und in der Versorgungspraxis wider [2].

Auch die Kinder- und Jugendmedizin, die Ende des 19. Jahrhunderts enormen Aufschwung genommen hat und zunächst stark sozialpädiatrisch geprägt war [3], hat im 20. Jahrhundert eine organbezogene Subspezialisierung erfahren. Die Unterteilung der Funktionsstörungen des Gehirns in zelluläre und strukturelle Störungen (Domäne der Neuropädiatrie) sowie seelische Störungen (Domäne der Kinder- und Jugendpsychiatrie) führte zu einer reduktionistischen Dichotomisierung von Erkrankungen in körperliche und seelische, mit der die Komplexität der Gesundheitsstörungen und Beeinträchtigungen der Kinder nur unvollständig abgebildet werden kann.

In den verschiedenen medizinischen und nicht-medizinischen Disziplinen sowie im Sozialrecht werden unterschiedliche Begriffe für körperliche, geistige oder seelische Abweichungen von der Gesundheit des einzelnen Menschen verwendet, die nicht immer synonym verwendet werden bzw. auch im Englischen unterschiedliche Entsprechungen haben. Sie werden in Tab. 1 dargestellt, da sie zum Verständnis der Literatur und der Versorgungspraxis von Bedeutung sind.

**Table 1:** 

Begriff	Bedeutung im alltäglichen Sprachgebrauch	Sozialrechtliche Nutzung des Begriffs	Terminologie nach ICD und ICF	Englische medizinische Begriffe
Körper (lateinisch „corpus“ = Körper, Leib)	Materielles Objekt, Physis des Menschen	SGB IX: Definition von Behinderung: *körperliche*, seelische, geistige oder Sinnesbeeinträchtigung Durchgängige Nutzung des Begriffs in allen Sozialgesetzbüchern (SGB)	ICD: bis auf Kap. F sind alle Gruppen bezogen auf körperliche Erkrankungen, meist organbezogen oder altersbezogen (Neonatologie)	Im Kontext Gesundheit immer „physical health“ ICF: „body functions“ und „body structures“
			ICF: Körperfunktionen sind die physiologischen Funktionen von Körpersystemen (*einschließlich psychologischer Funktionen*) Körperstrukturen sind anatomische Teile des Körpers, wie Organe und Gliedmaßen	
Soma/somatisch (griechisch „σῶμα*“* = Körper als Gegensatz zu Geist)	Körper/körperlich In der Biologie werden somatische (Körperzellen) von Keimbahnzellen unterschieden	SGB V: vereinzelte Nutzung des Begriffs: vgl. § 28 SGB V: „… Psychotherapeut [soll] vor Beginn der Behandlung den Konsiliarbericht eines Vertragsarztes zur Abklärung einer somatischen Erkrankung sowie, falls der **somatisch** abklärende Vertragsarzt dies für erforderlich hält, eines psychiatrisch tätigen Vertragsarztes einholen“	ICD 10: Kapitel F *somato*forme Störungen. Der Begriff wird hier ausschließlich genutzt, um körperliche Symptome bei psychischen Erkrankungen zu bezeichnen	Analog zu somatoformen Störungen „somatic symptom disorders“ z. B. in DSM‑5
Physis/physisch (griechisch „φύσις“ *=* natürliche Beschaffenheit als Gegensatz zur Psyche; s. unten)	Beschaffenheit des Körpers	Keine Verwendung	ICD 10: seltene Nutzung als physische Belastung (Z56, Z73, R53)	Häufige Nutzung im Sinne der World Health Organization (WHO)-Definition von Gesundheit: „physical, mental and social well-being“^a^
			ICF: durchgängig genutzt für „körperlich“	
Psyche/psychisch (griechisch „ψυχή“ *=* u. a. Seele, Geist, Gemüt)	Gesamtheit aller geistigen Eigenschaften, insbesondere Denken und Fühlen	Häufige Nutzung in SGB V und SGB IX (psychisch krank oder psychische Erkrankung); vgl. auch Aktion psychisch Kranke (APK) oder Modellprojekte für Kinder psychisch erkrankter Eltern	ICD 10: Kapitel F: Psychische und Verhaltensstörungen, klare Trennung von Kapitel G: Krankheiten des Nervensystems	„Psychological health“ – weitgehend synonym mit „mental health“, z. T. „emotional“, „behavioral“, und „psychosocial health“
			ICF: Doppelbegriff „seelisch/geistig“	
Seele (mittelhochdeutsch)	Immaterielle Innenwelt; i. d. R. verknüpft mit dem Leib, den sie nach dem Tod verlässt	SGB IX: Definition von Behinderung als körperliche, *seelische*, geistige oder Sinnesbeeinträchtigung	ICD 10: Kapitel F: geleg. Verwendung des Begriffs „seelisch“, z. B. O99, T74, F30	„Soul“, „spirit“, „mind“: keine Verwendung in der biomedizinischen Literatur
			ICF: In der deutschen Fassung wird der englische Begriff „mental“ meist mit dem Doppelbegriff „seelisch/geistig“ übersetzt	
Geist/geistig (indogermanisch) Auch als Gegensatz zu Körper und Seele als geistige Vorstellungen/Bewusstsein	Denkendes Bewusstsein des Menschen, Verstandeskraft, Verstand	SGB IX: Definition von Behinderung als körperliche, seelische, *geistige* oder Sinnesbeeinträchtigungen	ICD 10: im Wesentlichen keine Verwendung des Begriffs; Intelligenzstörungen im Kapitel F, dort geleg. Verwendung des Begriffs geistig, z. B. „geistige Retardierung“, auch in Q87, Z81	Mental (v. a. „mental health“) weitgehend synonym mit psychological health, schließt Kognition ein
			ICF: Doppelbegriff „seelisch/geistig“^a^	

*ICD* International Statistical Classification of Diseases and Related Health Problems, *ICF* International Classification of Functioning, Disability and Health, *DSM* Diagnostic and Statistical Manual of Mental Disorders

^a^*Die englische Definition der WHO von Gesundheit:* „Health is a state of complete physical, mental and social well-being“, wurde als „Gesundheit ist der Zustand des vollständigen körperlichen, geistigen und sozialen Wohlbefindens“ ins Deutsche übertragen. In der deutschen Fassung der ICF wird demgegenüber das Wort „mental“ immer als Begriffspaar seelisch/geistig geführt. Diese Nomenklatur wurde weitgehend in das SGB IX übernommen

Wir verwenden in diesem Beitrag den Begriff *mental* als Summe aller kognitiven und psychischen Prozesse (analog der Übersetzung von mental als geistig/seelisch in der International Classification of Functioning, Disability and Health – ICF) und den Begriff *körperlich* als Summe aller anderen Prozesse, die das Leben unterhalten. Dies folgt den internationalen Klassifikationssystemen, aber nicht einem aus unserer Sicht veralteten, im Sozialrecht verfestigten Begriff von Behinderung, der körperliche, seelische, geistige oder Sinnesbeeinträchtigungen unterscheidet, was unterschiedliche Einschätzungs- und Versorgungsprozesse nach sich zieht (Tab. 1).

Die Steuerung aller körperlichen Prozesse geht vom zentralen Nervensystem aus, ebenso wie die Gestaltung mentaler Prozesse. Beides ist untrennbar verbunden: mentale Prozesse veranlassen, begleiten und speichern Abläufe der Bewegung, der Immunantwort, der Hormonausschüttung (um nur einige Aspekte plakativ zu benennen) und körperliche Prozesse begleiten, modifizieren und unterhalten mentale Prozesse. Dabei kommt es im Rahmen der neurobiologischen Entwicklung des Gehirns, deren bedeutendster Abschnitt sich über die pränatalen und frühen Kindheitsjahre erstreckt, überhaupt erst zur Differenzierung zwischen mentalen und körperlichen Prozessen auf der Ebene der sensorischen Verarbeitung, motorischen Antworten und der Entstehung von Gefühlswahrnehmungen. Die Unterscheidung von mentalen und körperlichen Erkrankungen, insbesondere bei jungen Kindern, ist oft sehr schwierig. Die heilkundliche (Differential)diagnostik und Klärung der Ätiologie der Funktionsstörung des Körpers verbessern die Voraussetzung für individuelle Prävention, Therapie oder Rehabilitation. Diese kann eingebettet werden in eine ganzheitliche Sicht, die dem betroffenen Menschen ein individuelles Krankheitsverständnis und eine salutogenetische Bewältigung der Herausforderung erlauben.

Zur Veranschaulichung werden in diesem Beitrag exemplarisch einige angeborene oder früh erworbene Störungen dargestellt, die durch Abweichungen von der typischen körperlichen und mentalen Entwicklung gekennzeichnet sind. Die Folgen exogener Noxen, transgenerationaler Einflüsse und belastender Kindheitserfahrungen (Adverse Childhood Experiences – ACE) sowie das gemeinsame Auftreten körperlicher, mentaler und sozialer Einflussfaktoren werden geschildert, um die komplexen Interaktionen darzustellen. Die Bedeutung einer Verwirklichung des biopsychosozialen Verständnisses in der alltäglichen gesundheitlichen Versorgung wird im Hinblick auf die Behandlung der Kinder und ihrer Familien verdeutlicht. Abschließend werden Vorschläge für eine verbesserte Versorgung von Kindern und Jugendlichen mit komplexen chronischen Gesundheitsstörungen durch praktische Interdisziplinarität gemacht.

## Das Gehirn: Entwicklung und Funktion

Bis zum Alter von 5 Jahren entwickelt sich das Gehirn eines Kindes mehr als zu jedem anderen Zeitpunkt im Leben, weshalb wir häufig von einem „sensitiven/kritischen“ Zeitfenster sprechen. Während die Neuronen bereits in der Pränatalzeit der menschlichen Hirnentwicklung gebildet werden, finden postnatal vor allem Synaptogenese und Myelinisierungsprozesse statt. Das Hirnvolumen verdoppelt sich im ersten Lebensjahr, im Alter von 5 Jahren hat es bereits 90 % des adulten Volumens erreicht [4].

Die Entwicklung der neuronalen Schaltkreise des Gehirns erfordert die Koordination einer außerordentlich komplexen Reihe von Ereignissen der Neuroentwicklung [5]. Beginnend in der Pränatalzeit wird sie von vielen Faktoren beeinflusst: Neben genetischen Prädispositionen spielen insbesondere frühe Umwelteinflüsse, wie z. B. die Qualität und Quantität der Interaktionen mit frühen Bezugspersonen, und neuroplastische Reaktionen auf verschiedene Reize eine große Rolle.

Neue Erkenntnisse belegen, dass das Darmmikrobiom Einfluss auf die Neurogenese, Myelinisierung und Mikroglia-Reifung nimmt und sowohl mit der Entwicklung und Aufrechterhaltung der Integrität der Blut-Hirn-Schranke als auch mit der Entwicklung der Hypothalamus-Hypophysen-Nebennierenrinden-Achse (HPA-Achse) und deren Stressreaktion zusammenhängt. Während der fetalen Entwicklung fällt die Erstbesiedlung des Mikrobioms mit der Entwicklung des Nervensystems in einer zeitlich abgestimmten Weise zusammen. Die Zusammensetzung und Vielfalt des Darmmikrobioms, die Genetik, mütterliche und andere Faktoren können die gesamte Entwicklungshomöostase verändern, indem sie Störungen des Immunsystems verursachen. Darmdysbiose, Immunveränderungen und andere Faktoren führen über eine Entzündungsreaktion zu einer Aktivierung der Mikroglia, was zu einer systemischen Entzündung führen kann. Dies wirkt sich negativ auf den Entwicklungsprozess des Gehirns aus und kann eine dysfunktionale Gehirnentwicklung und -funktionalität bedingen [6].

## Angeborene Störungen

Bei abweichender Entwicklung oder bei Erkrankungen des Gehirns sind meistens mehrere neurologische Funktionen beeinträchtigt, mit Auswirkungen auf verschiedene Aktivitäten des Kindes. Neben sichtbaren körperlichen Dysmorphien besteht häufig ein charakteristischer Verhaltensphänotyp.

**Figure 1:**
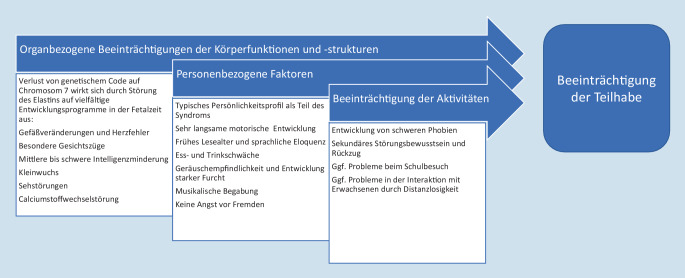

Als Beispiel für zahlreiche andere genetische, syndromale Erkrankungen, aber auch ätiologisch unklare Fehlbildungs‑/Retardierungssyndrome soll das Williams-Beuren-Syndrom kurz beschrieben werden (Abb. 1). In der Keimbahnentwicklung kommt es hierbei zu einem Verlust des Elastin-Gens und weiterer benachbarter Gene auf dem Chromosom 7, was sich auf die Bildung von Bindegewebe auswirkt. Dass dies zu einer Schwäche der Muskulatur, auch im Gefäßsystem, und damit Herzfehlern und zu Kleinwuchs führen kann, mag sofort einleuchten. Aber die Pathogenese immunologischer Störungen mit vermehrter Infektneigung oder endokrinologischer Auffälligkeiten wie eine Hypercalcämie beim Williams-Beuren-Syndrom sind ebenso unklar wie die Entstehung bestimmter Persönlichkeitsmerkmale wie Musikalität oder Furchtsamkeit [7, 8].

Als Beispiel für die Auswirkungen exogener Noxen für die frühe Gehirnentwicklung sei Alkohol genannt. Alkoholkonsum in der Schwangerschaft ist eine der häufigsten Ursachen einer Entwicklungsstörung. Alkohol führt zu einer pränatalen Schädigung der körperlichen und mentalen Funktionen durch komplexe Pathomechanismen wie Apoptose, Modulation von Genexpression und Störungen neuronaler Migration/Axonenbildung. Die Folge sind körperliche Symptome wie ein mangelhaftes Wachstum von Körper und Gehirn, faziale Auffälligkeiten und mentale Symptome durch eine Störung der neuronalen Konnektivität (z. B. in den Bereichen Gedächtnis, Exekutivfunktionen, Sinnesbeeinträchtigungen, Verhaltensauffälligkeiten) und durch Probleme in der sozialen Adaptation [9].

Zu den genetischen oder exogen-toxischen Störungen kommen soziale und umweltbezogene Faktoren. Die Variabilität von Entwicklungsverläufen ist bei Kindern mit angeborenen syndromalen Erkrankungen oder exogen-toxischen Entwicklungsstörungen ebenso groß wie bei Kindern ohne erkennbare Beeinträchtigungen. Allerdings müssen Kinder mit gesundheitlichen Einschränkungen anstehende Entwicklungsaufgaben immer vor dem Hintergrund der vorhandenen Beeinträchtigungen bewältigen. Dies kann besondere Probleme bereiten, wenn das mentale Funktionsniveau und damit die Anpassungsbereitschaft gering sind und/oder wenn die Zahl der äußeren sozialen und umweltbezogenen Belastungen hoch ist [10].

## Entzündliche Ursachen akuter neurologischer und psychiatrischer Erkrankungen

Inflammatorische Erkrankungen des Gehirns können zu vielfältigen mentalen und körperlichen Symptomen führen und sollen in diesem Beitrag exemplarisch darauf hinweisen, dass durch neue molekularbiologische und immunologische Erkenntnisse die Trennung von körperlichen und mentalen Krankheitsprozessen kaum möglich ist.

Als erstes Beispiel sei hier eine 2007 von der Arbeitsgruppe Dalmau beschriebene Form der limbischen Enzephalitis angeführt, die durch NMDA-Rezeptor^1^-Antikörper verursacht wird [11]. Zu Beginn der Erkrankung finden sich häufig grippeartige, gefolgt von neuropsychiatrischen Symptomen (akute Psychose, Verhaltensstörungen, Gedächtnisstörungen), Bewegungsstörungen und/oder epileptischen Anfällen sowie einer autonomen Regulationsstörung. Die Autoantikörper führen zu einer Herunterregulation des NMDA-Rezeptors im Bereich der Synapsen. Die unterschiedlichen Symptome konnten inzwischen teilweise der Fehlfunktion einzelner Hirnstrukturen bzw. neuronalen Netzwerken zugeordnet werden, so z. B. Gedächtnisstörungen den hippocampalen Netzwerken und psychotische Symptome den frontoparietalen Netzwerken [12]. Während dieses Krankheitsbild heutzutage meist gut durch eine immunmodulatorische Therapie zu behandeln ist, war dies vor der Entdeckung der autoimmunologischen Genese nicht der Fall. Das Krankheitsbild wurde als ein rein psychiatrisches angesehen und zeigte häufig eine medikamentös schwierig zu behandelnde Psychose mit einem prolongierten und wiederkehrenden Verlauf [13].

Als zweites Beispiel sei die Erkrankung ME/CFS (myalgische Enzephalitis/chronisches Fatigue-Syndrom) angeführt, welche ebenfalls zu vielfältigen körperlichen und mentalen Symptomen führt. Die Pathophysiologie dieser Erkrankung ist bislang nicht vollständig verstanden. Sie tritt nach bestimmten Virusinfekten auf und ist bislang nicht ursächlich zu behandeln. Die Therapie besteht auf der einen Seite aus medikamentösen Ansätzen einer symptomorientierten Behandlung von körperlichen Beschwerden, auf der anderen Seite aus nicht-medikamentösen Behandlungen zur Besserung körperlicher und mentaler Symptome, wie z. B. dem Erlernen von Entspannungstechniken und dem sogenannten Pacing (Methode zur Überlastungsvermeidung) sowie einer kognitiven Verhaltenstherapie [14]. Eine Nichtbeachtung der komplexen biopsychosozialen Faktoren dieser Erkrankung führt zu einem schlechteren Verlauf.

## Transgenerationale Aspekte in der Regulation der neuronalen, endokrinen und immunologischen Entwicklung

Während der Gehirnentwicklung genießen die stattfindenden Wachstums- und Differenzierungsprozesse eine Priorität in der Versorgung mit Energiestoffen gegenüber anderen Organen. Das menschliche Gehirn verfügt über entsprechende Steuerungsprozesse [15]. Es wird vermutet, dass im Hypothalamus über empfindliche Sensoren die Zufuhr von Blutglukose ins Gehirn gesteuert wird, und zwar in einer Weise, dass die Energieversorgung des Gehirns nicht gefährdet wird. Diese Mechanismen tragen dazu bei, dass es bei einer intrauterinen Mangelernährung (z. B. bei Fehl- oder Unterernährung der Schwangeren, schwerem psychosozialen Stress oder starkem Nikotinabusus) häufig zu starkem Untergewicht des Neugeborenen, jedoch in weit geringerem Umfang zu einer Minderung des Kopfumfangs kommt. Die prioritäre Zufuhr von Glukose zum Gehirn führt jedoch auch dazu, dass in chronischen Stresssituationen dauerhaft die Bereitstellung von Glukose im Körper erhöht ist, was im späteren Leben trotz Untergewicht bei der Geburt zu einem metabolischen Syndrom mit Übergewicht, Bluthochdruck und kardiovaskulären Schäden, aber auch mentalen Erkrankungen wie Depressionen führen kann [16]. Der Hungerwinter 1944/1945 und die transgenerationalen Folgen für die Kinder der damals schwangeren Frauen werden durch epigenetische Veränderungen erklärt [17].

Diese Beispiele gehören zu den eindrucksvollsten Erkenntnissen über die sog. fetale Programmierung („fetal programming“) und zeigen, dass bereits frühe Einflüsse der Umwelt, die intrauterin auf den Fetus wirken, Folgen für das gesamte weitere Leben haben können [18]. Auch eine prä- oder postnatale antibiotische Therapie als exogene Umweltfaktoren wurden beispielsweise mit veränderten Raten von Adipositas assoziiert, die durch eine Veränderung des Mikrobioms vermittelt sein könnten [19].

Im Zusammenhang mit der Stressregulation entsteht häufig auch die Neigung zu einer Essstörung und Entwicklung einer frühen Adipositas, die oft sowohl bei Mutter wie auch dem Kind besteht. Trotz vermehrter gesundheitsfördernder Anstrengungen konnte die Prävalenz von Adipositas durch verhaltenstherapeutische Ansätze in psychosozial belasteten Familien nicht gesenkt werden [20]. Im Gegenteil haben sich die sozialen Unterschiede in der Verbreitung von Übergewicht im Kindes- und Jugendalter seit Anfang der 2000er Jahre weiter ausgeweitet, sodass sich die Auswirkung der Ausgrenzung von sozial benachteiligten Familien verstärken [21]. Adipositas ist dabei zunehmend selbst zu einem Anlass für weitere Stigmatisierung geworden. Schließlich führt die Kombination aus Adipositas und den vielfältigen begleitenden mentalen Beeinträchtigungen auch zu sozialem Misserfolg und mangelndem Bildungserfolg [22]. Ein breiteres Verständnis und interdisziplinärer Austausch sind für die Entwicklung wirksamer und passgerechter Interventionen notwendig, wenn die hohen gesellschaftlichen und individuellen Folgekosten und Leiden bei dieser komplexen Essstörung vermieden werden sollen.

## Wechselwirkungen körperlicher, mentaler und sozialer Determinanten

Die enge Verknüpfung mentaler und körperlicher Beeinträchtigungen zeigt sich auch in der Epidemiologie der Komorbidität psychischer Beeinträchtigungen bei primär körperlichen chronischen Erkrankungen. Während in der Gruppe der Kinder und Jugendlichen ohne erkennbare körperliche Erkrankung in Screening-Verfahren die Häufigkeit von psychischen Beeinträchtigungen zwischen 15 % und 20 % geschätzt wird [23], liegt sie bei Kindern und Jugendlichen etwa doppelt so hoch, insbesondere bei Erkrankungen mit neurologischen Funktionsstörungen. Dies gilt auch, wenn nur Kinder und Jugendliche mit klinischen Diagnosen betrachtet werden [24]. Die therapeutischen Ansätze bei Auftreten von psychischen Störungen und gleichzeitig bestehenden körperlichen Erkrankungen sind sehr unterschiedlich und richten sich nach der individuellen Befundkonstellation [25, 26].

Umgekehrt ist auch die Prävalenz körperlicher Erkrankungen bei Kindern und Jugendlichen mit psychischen Störungen deutlich erhöht. So zeigte beispielsweise eine schwedische Registerstudie, dass Kinder und Jugendliche mit Angst- und Verhaltensstörungen in fast allen Altersgruppen eine deutlich höhere Rate an körperlichen Erkrankungen aufwiesen als Kinder ohne psychische Störungen. Affektive Störungen konnten mit verschiedensten körperlichen Erkrankungen im Alter von 12–18 Jahren in Verbindung gebracht werden, bei psychotischen Störungen im Jugendalter zeigten sich vermehrt Asthma, Darmerkrankungen und Myalgien [27]. Auch in Hinblick auf Anorexia nervosa mehren sich die Hinweise, dass diese als eine metabolisch-psychiatrische Erkrankung zu verstehen ist, bei der auch körperliche Faktoren zur Aufrechterhaltung der Essstörung beitragen. Neben dem direkten Einfluss des Mikrobioms auf das Gehirn und dem Verhalten der Betroffenen spielen auch eine veränderte Energieaufnahme aus der Nahrung, hormonelle Veränderungen, eine wahrscheinlich erhöhte Durchlässigkeit des Darms sowie entzündliche und immunologische Prozesse eine wichtige Rolle [28].

Im Einzelfall ist zu klären, ob es sich bei einer psychischen Beeinträchtigung um eine sekundäre Entwicklung aufgrund negativer persönlicher oder sozialer Erfahrungen von Diskriminierung und Exklusion handelt, um einen Teil des primären Störungsbildes oder um ein Resultat weiterer Risikofaktoren. Vor allem bei einer chronischen Gesundheitsstörung im Kindes- und Jugendalter ist es daher wichtig, einen vollständigen körperlichen wie auch psychischen Befund zu erheben, eine Sozial- und Familienanamnese durchzuführen, vorliegende Befunde aus der Krankengeschichte zu würdigen sowie ggf. weitere Expertise hinzuzuziehen [29].

In Bezug auf äußere soziale Kontextfaktoren besteht im Rahmen der neurobiologischen Entwicklung auch jenseits der Fetalzeit eine besondere Vulnerabilität. Zu den belastenden Kindheitserfahrungen (ACE) gehören Erfahrungen von frühkindlicher Deprivation, Gewalt durch Bezugspersonen, sexuellem Missbrauch, „toxischem Stress“, Diskriminierung und Stigmatisierung, ausgeprägter Armut und Nahrungsmittelunsicherheit sowie das Leben in Familien oder Nachbarschaften mit einer hohen Rate von Kriminalität und Dissozialität [30]. In der Folge solcher Erfahrungen ist ein deutlich gehäuftes Auftreten von psychischen Erkrankungen zu beobachten, insbesondere von Depression, Angsterkrankungen, Essstörungen, Abhängigkeitserkrankungen und vermehrten Suiziden. Jedoch konnten Studien auch Auswirkungen auf den allgemeinen Gesundheitszustand, funktionelle Einschränkungen, Diabetes, kardiovaskuläre und immunologische Erkrankungen nachweisen [31–33]. Vermutlich spielen bei der Entstehung der körperlichen Folgen miteinander verknüpfte Mechanismen eine Rolle: 1.) Beeinträchtigung der Stressregulation und chronisch erhöhte entzündliche Reaktion durch die ACE, 2.) durch die Lebensumstände vermehrt auftretende Verhaltensmuster wie Impulsivität, veränderte soziale Wahrnehmung und negative Zukunftserwartungen und 3.) riskantes Gesundheitsverhalten [34].

Diese Mechanismen erklären auch die doppelte Gefahr, die durch das Aufwachsen in deprivierten Nachbarschaften, Gewalterfahrung und mangelnde Beziehungskontinuität sowohl für Mütter als auch für ihre Kinder besteht, Beeinträchtigungen der mentalen Funktionen zu erleiden und dem Teufelskreis mit schlechteren Gesundheitschancen nicht entkommen zu können. Andererseits bietet sich hier die Möglichkeit für Interventionen im Hinblick auf veränderbare Faktoren in der Lebenswelt der Kinder [35].

## Vermittlung von Diagnosen und Hypothesen an betroffene Eltern

Die Pflege, Erziehung und Förderung eines Kindes mit einer chronischen körperlichen und/oder psychischen Gesundheitsstörung können die Eltern und die ganze Familie in besonderer Weise belasten. Die familiären Interaktionen können zudem die Symptomatik und die Schwere der Beeinträchtigung des Kindes und das Wohlbefinden aller Familienmitglieder positiv oder negativ beeinflussen [36]. Wenn der Nachweis einer objektivierbaren Funktionsstörung fehlt, stützt dies nicht die Annahme eines ätiologischen Zusammenhangs mit dem elterlichen Erziehungsverhalten.

Etwa die Hälfte aller angeborenen Fehlbildungs- und Entwicklungsstörungen können heute noch nicht diagnostisch geklärt werden. Zudem werden die Störungen, insbesondere bei Fehlen ausgeprägterer Dysmorphien, oft nicht vermutet und damit auch keiner Klärung zugeführt.^2^ Die Erfahrungen von Eltern im diagnostischen Prozess einer entwicklungs-, verhaltens- oder schulbezogenen Störung sollten umsichtig wahrgenommen und anerkannt werden, um weitere Belastungen der Eltern zu vermeiden. Dies gelingt, wenn nicht nur die augenblickliche Symptomatologie (z. B. Wutanfälle, Unruhe, Essstörung) in den Blick genommen wird, sondern eine biographische Anamnese bezüglich der Säuglingszeit und eine umfassende Familienanamnese erhoben werden.

Bei einem positiven (auffälligen) genetischen Befund können die gefundenen Genveränderungen oft nicht als alleinige Ursache der gesundheitlichen Störung ausgewiesen werden. Sogar bei den bislang über 2000 aufgeklärten monogenetischen Erkrankungen (Ein-Gen-Erkrankungen) wie Hämophilie, Muskeldystrophie Duchenne, Phenylketonurie oder zystische Fibrose kann eine große Variabilität in der Ausprägung bestehen, weil in einem einzigen Gen Hunderte verschiedene Mutationen auftreten können. Dennoch lässt sich eine enge kausale Beziehung zwischen dem Genotyp und dem klinischen Phänotyp ausmachen. Bei polygenetischen Erkrankungen ist der Phänotyp jedoch sehr viel häufiger abhängig von einer unbekannten Anzahl weiterer Entwicklungsgene sowie den oben beschriebenen Einflussfaktoren, die über neuronale, endokrinologische oder immunologische Mechanismen vermittelt werden. Die Kenntnis der genetischen Veränderungen und der Systemmodulation ist von großer Bedeutung. So können immer mehr kausale Therapien entwickelt werden, die direkt an den Gendefekten oder an modulatorischen Prozessen angreifen.

**Figure 2:**
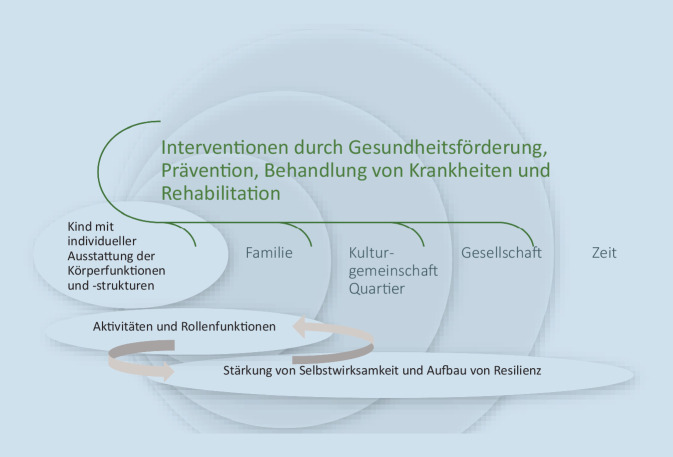

Therapeutisch und im Umgang mit Familien muss das Bedürfnis nach einem monokausalen Erklärungsmuster auf beiden Seiten wahrgenommen und kritisch reflektiert werden. Kausale Prozesse sind lineare Prozesse, angemessener erscheint das Verständnis eines Krankheitsprozesses als Kreislauf mit selbstverstärkenden negativen und positiven Einflüssen. Auch ohne Kenntnis der genauen Ätiopathogenese bietet solch ein Verständnis die Möglichkeit, gemeinsam mit den Betroffenen den problematischsten Aspekt im Teufelskreis unter Mitwirkung aller anzugehen und damit zu versuchen, eine positive Entwicklung anzustoßen und die negativen Verstärker zu reduzieren. Häufig sind die Kinder und Jugendlichen sowie die Eltern die Experten im Verständnis der Erkrankung; die Beachtung ihrer Behandlungsprioritäten und Unterstützungswünsche kann ihre Mitwirkung und Selbstwirksamkeit erheblich verstärken. Es sollte keinen prinzipiellen Unterschied machen, ob die Symptomatologie überwiegend im Bereich der mentalen oder der körperlichen Prozesse angesiedelt ist, Ziel ist die Förderung von aktiver Lebensgestaltung und Teilhabe [37]. Dieser teilhabeorientierte Behandlungsansatz wird in Deutschland in der gesundheitlichen Versorgung von Kindern und Jugendlichen mit komplexen Gesundheitsstörungen insbesondere in den sozialpädiatrischen Zentren und Kliniken verfolgt, zunehmend auch in Spezialambulanzen für Kinder und Jugendliche mit körperlichen chronischen Erkrankungen sowie der kinder- und jugendpsychiatrischen Versorgung, soweit dort Kinder und Jugendliche mit körperlichen Erkrankungen betreut werden. Immer erfolgt die Behandlung dann interdisziplinär bzw. multiprofessionell und folgt einem patientenorientierten und partizipativen Ansatz ([38]; Abb. 2).

## Fazit und Empfehlungen

Der noch häufig sowohl in der Kinder- und Jugendmedizin als auch in der Kinder- und Jugendpsychiatrie anzutreffende Dualismus zwischen „psychischen“, d. h. mentalen Beeinträchtigungen und „somatischen“, d. h. körperlichen Beeinträchtigungen sollte kritisch und fachübergreifend diskutiert werden. Es ist hervorzuheben, dass die International Classification of Functioning, Disability and Health (ICF) die mentalen, d. h. auch die psychischen Funktionen dem Körper zuordnet: „Der Begriff ‚Körper‘ bezieht sich auf den menschlichen Organismus als Ganzes. Daher umfasst er auch das Gehirn und seine Funktionen. Aus diesem Grund werden mentale (geistige und seelische) Funktionen unter ‚Körperfunktionen‘ subsumiert“ [39]. Der Wissenszuwachs über das Zusammenspiel von genetischen, neurologischen, immunologischen, endokrinologischen und geistig/seelischen Prozessen verlangt nach einer neurobiologisch fundierten Psychosomatik unter starker Berücksichtigung von sozialen und Umweltfaktoren [1].

Der Dualismus Körper und Geist entspricht vermutlich einem Bedürfnis nach Reduktion der Komplexität und monokausalen Erklärungsmustern. Dem zunehmenden Spezialwissen und der damit verbundenen Kompartimentierung des Wissens bei gleichzeitigem Wunsch nach einem ganzheitlichen Krankheits- und Gesundheitsverständnis kann Rechnung getragen werden durch eine nachhaltige Struktur der Interdisziplinarität. Wir möchten 3 Lösungen vorschlagen:

1. Die Aus‑, Weiter- und Fortbildung in den kinder- und jugendmedizinischen Fächern, sowohl den konservativen und operativen als auch den psychiatrischen Fachrichtungen, sollte vermehrt Gelegenheit zum interdisziplinären Lernen und Erkenntnisgewinn bieten.
2. Generische und dem biopsychosozialen Modell von Krankheit und Gesundheit folgende Begrifflichkeiten wie „chronische Gesundheitsstörungen“, „Beeinträchtigungen von Funktionen“, „Behindert-Werden“ und „Teilhabestörungen“ können helfen, Abgrenzungen zu vermeiden, wo sie im Sinne der Versorgungspraxis oder der Beschreibung der Kinder- und Jugendgesundheit nicht hilfreich sind.
3. Eine gemeinsame Verständigung in den konservativen und operativen kinder- und jugendmedizinischen und -psychiatrischen Fächern kann die gemeinsame Sprache im Hinblick auf die Umsetzung der Zusammenführung der Kinder- und Jugendhilfe und der Eingliederungshilfe unterstützen. Dies ist eine wesentliche Voraussetzung dafür, tatsächlich eine Inklusion mit dem Ziel einer gleichberechtigten Teilhabe unabhängig von Störungsbildern oder Behinderungsarten zu erreichen [40].

## Footnotes

1 NMDA-Rezeptor: gehört zu den ionotropen Glutamatrezeptoren, kommt v. a. im Zentralnervensystem vor, wurde nach dem ebenfalls wirksamen selektiven Agonisten N‑Methyl-D-Aspartat benannt.

2 Der Bericht einer Mutter, bei deren Tochter das Williams-Beuren-Syndrom erst im jungen Erwachsenenalter diagnostiziert wurde, zeigt die Belastungen und den fehlenden Zugang zu Unterstützung. Helios Magazin Mai 2020. Seltene Erkrankung: Williams-Beuren-Syndrom. (Zitierdatum 20.12.2022) Aufrufbar unter https://www.helios-gesundheit.de/magazin/echte-momente/news/seltene-erkrankung-williams-beuren-syndrom/.
